# Concurrent treatment with rituximab and plasma exchange for severe refractory granulomatosis with polyangiitis: A case report

**DOI:** 10.1097/MD.0000000000018139

**Published:** 2019-12-20

**Authors:** Ran Song, Sang Wan Chung, Yeon-Ah Lee

**Affiliations:** Department of Rheumatology, School of Medicine, Kyung Hee University, Seoul, South Korea.

**Keywords:** case report, granulomatosis with polyangiitis, plasma exchange, rituximab

## Abstract

**Rationale:**

Rituximab is recommended to induce remission of severe granulomatosis with polyangiitis (GPA). Plasma exchange (PE) may be considered in the setting of rapidly progressive glomerulonephritis (RPGN) with a serum creatinine increase of more than 5.6 mg/dl or diffuse alveolar hemorrhage (DAH). However, there are no sufficient studies on combination therapy with rituximab and PE in GPA.

**Patient concerns:**

A 23-year-old woman was admitted with fever, abdominal pain, and diarrhea on suspicion of infectious colitis. Colonoscopy showed hemorrhagic colitis and antibiotic treatment was ineffective. Physical examination revealed episcleritis and skin lesions similar to Janeway lesions or Osler nodes on her palms and soles. Transesophageal echocardiogram (TEE) revealed mitral valve vegetation mimicking infective endocarditis. However, no pathogen was grown in the blood culture. Ten days after admission, blood-tinged sputum and respiratory distress developed. Imaging studies of lung, bronchoscopy, and bronchoalveolar lavage indicated DAH. Moreover, serum creatinine levels rapidly increased from 0.8 mg/dl to 6.1 mg/dl with proteinuria.

**Diagnosis:**

The patient was diagnosed with GPA and non-infectious endocarditis, DAH, and RPGN, based on a biopsy which revealed pauci-immune crescentic glomerulonephritis with granuloma and leukocytoclastic vasculitis and antineutrophil cytoplasmic antibodies against proteinase 3- positivity.

**Interventions:**

Initial methylprednisolone pulse therapy (1 g daily for 3 days) proved unsuccessful. After initiating PE, creatinine levels began to slowly decline, but DAH continued to deteriorate. Rituximab combined with PE therapy was considered. We performed PE every 2 to 3 days for 5 total treatments combined with rituximab (375 mg/m^2^, once weekly for 4 weeks).

**Outcomes:**

After the combination treatment of rituximab and PE, alveolar hemorrhage stopped. Chest X-ray and laboratory data, including serum creatinine and hemoglobin, notably improved. Mitral valve vegetation was no longer observed in follow-up TEE. GPA remained stable with low dose prednisolone and immunosuppressants over a follow-up period of 5 years.

**Lessons:**

This case suggests that the use of rituximab and concurrent PE may represent a promising combination for severe and refractory GPA.

## 1 Introduction

Granulomatosis with polyangiitis (GPA) is a necrotizing vasculitis of the medium and small blood vessels, which can be fatal. Without proper treatment, approximately 80% of cases do not survive more than a year.^[1]^ In particular, rapidly progressive glomerulonephritis (RPGN) or diffuse alveolar hemorrhage (DAH) are well-known life-threatening conditions in GPA. Rituximab is a chimeric monoclonal antibody directed against CD20 and recommended for the induction of remission in severe active GPA.^[2,3]^ Plasma exchange (PE) may be considered for treating RPGN when the serum creatinine level is >5.6 mg/dl or in the setting of life-threatening DAH.^[4,5]^ Concurrent use of rituximab and PE has been reported in antibody-mediated rejection after kidney transplantation or in thrombotic thrombocytopenic purpura. However, no case reports or studies have discussed the efficacy of rituximab plus concurrent plasma exchange for treating GPA. We report a case of GPA with diffuse non-infectious endocarditis, DAH, and RPGN, which was treated successfully with rituximab and PE combination therapy.

The institutional review board approved the study. Written informed consent was obtained from the patient for publication of the case.

## 2 Case presentation

**Figure 1:**
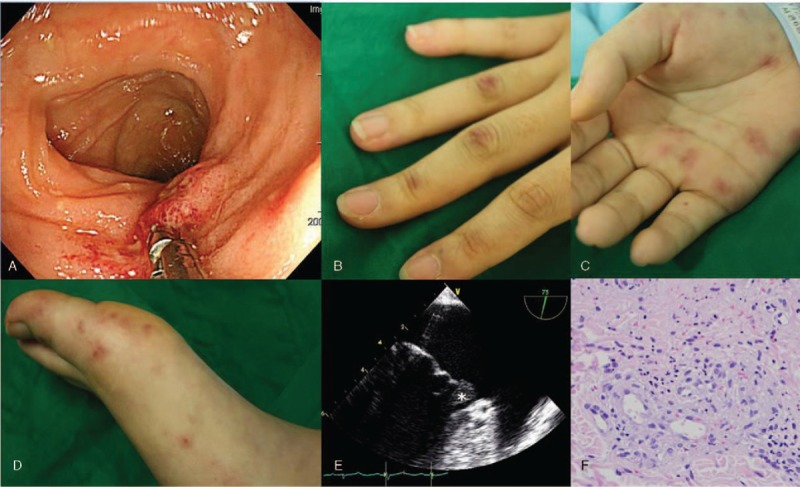
Clinical manifestations and pathologic findings. A. Colonoscopy showing a 1 cm aphthous ulcer at the terminal ileum and multiple reddish spots on the entire colon. B. Erythematous macules on fingers. C. Violaceous subcutaneous nodules on the palm. D. Erythematous papules and violaceous subcutaneous nodules on the sole. E. Vegetation of the mitral valve (white asterisk) with transesophageal echocardiogram. F. Skin biopsy showing diffuse perivascular chronic inflammatory cell infiltration. (H&E stain, original magnification, ×400).

A 23-year-old woman presented to our emergency room with fever, abdominal pain, vomiting, and diarrhea, which manifested after eating raw fish 3 days ago. Laboratory examination revealed mild anemia (hemoglobin (Hb) 11.5 g/dl) and elevated levels of erythrocyte sedimentation rate (ESR) (74 mm/hour) and C-reactive protein (CRP) (13.79 mg/dl). Blood chemistry results, including renal function, were within reference ranges. Abdominal computed tomography (CT) showed no specific findings except heterogeneous enhancement of the spleen. Colonoscopy showed hemorrhagic colitis (Fig. 1A) and she was admitted on suspicion of infectious colitis. Administration of intravenous antibiotics was ineffective. Three days after admission, the patient developed episcleritis and erythematous macules and violaceous subcutaneous nodules on the fingers (Fig. 1B), palms (Fig. 1C), and soles (Fig. 1D) which appeared similar to Janeway lesions or Osler nodes. Transesophageal echocardiogram (TEE) revealed mitral valve vegetation with mild regurgitation mimicking infective endocarditis (Fig. 1E). However, the blood culture did not yield any pathogens. The skin biopsy showed diffuse perivascular chronic inflammatory cell infiltration consistent with leukocytoclastic vasculitis (Fig. 1F).

**Figure 2:**
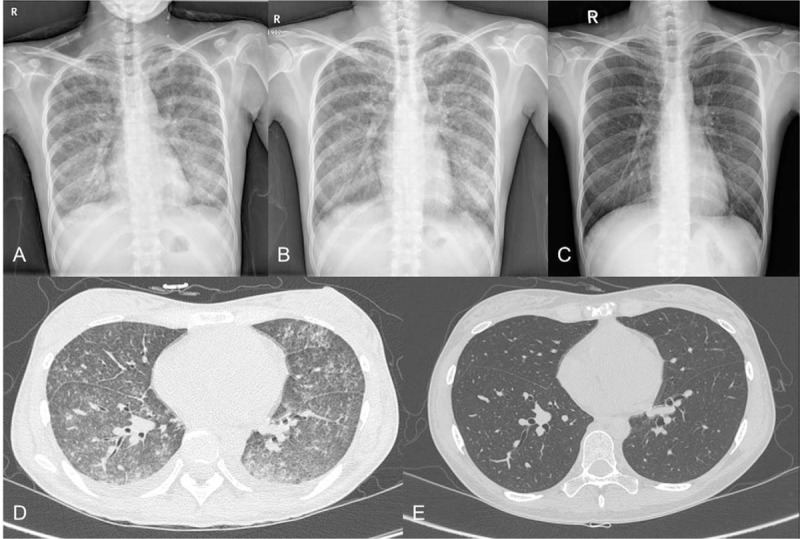
Imaging findings during deterioration and improvement of diffuse alveolar hemorrhage. A. Chest X-ray showing reticular opacity in the whole lung field following the development of respiratory distress. B. Chest X-ray showing no significant changes with plasma exchange alone. C. Chest X-ray showing marked improvement in diffuse lung infiltration after combination therapy with rituximab and plasma exchange. D. Chest CT revealing diffuse ground-glass opacity in both lung fields. E. Chest CT showing improvement in diffuse ground-glass opacity with interlobular septal thickening.

**Figure 3:**
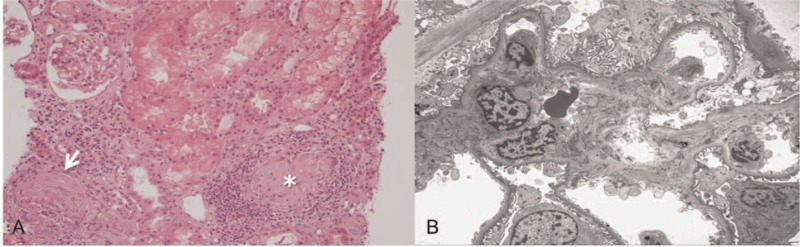
Pathologic findings of renal biopsy. A. Light microscopy (hematoxylin and eosin (H&E) stain, original magnification, ×400) showing glomerulitis with the crescent formation (arrow) and periglomerular granuloma (asterisk). Mononuclear cell infiltration and interstitial fibrosis with tubular atrophy were observed in the tubulointerstitium. B. Electron microscopic (original magnification, ×400) finding showing focal foot process effacement and increased mesangial matrix and cells.

**Figure 4:**
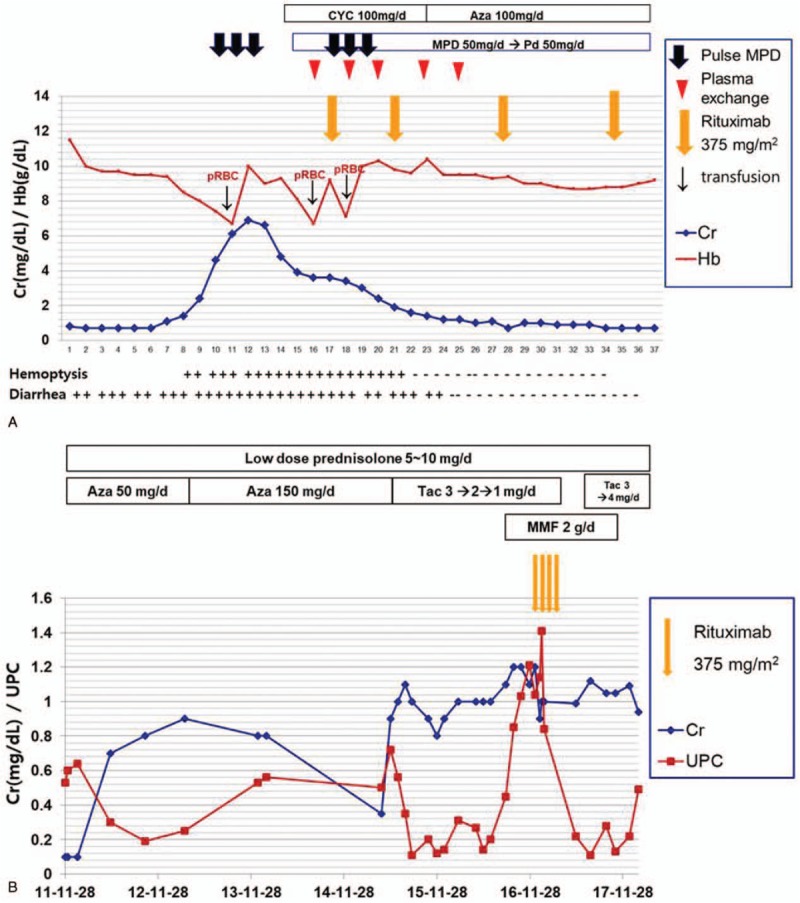
Clinical course during the first admission (A) and changes in kidney function during the long-term follow-up period (B). A. Dosing schedules of rituximab and plasma exchange and changes in creatinine and hemoglobin levels. Plasma exchanges were performed every 2 to 3 days for a total of 5 treatments (days 16, 18, 20, 23, and 25), combined with rituximab (once weekly for 4 weeks). B. Changes in creatinine and UPC levels during the 6-year follow-up period. Aza = azathioprine, Cr = creatinine, CYC = cyclosporine, Hb = hemoglobin, MMF = mycophenolate mofetil, MPD = methylprednisolone, Pd = prednisolone, pRBC = packed red blood cell, Tac = tacrolimus, UPC = urine protein to creatinine ratio.

Ten days after admission, the patient presented with blood-tinged sputum and respiratory distress (respiratory rate, 44 breaths/minute; oxygen saturation, 90% on 1 L/minute oxygen). The Hb level abruptly declined to 6.4 g/dl. Chest imaging showed reticular opacity on X-ray (Fig. 2A) and diffuse ground-glass opacity in both lung fields on CT (Fig. 2D). Bronchoscopy and bronchoalveolar lavage indicated DAH. Moreover, serum creatinine levels rapidly increased from 0.8 to 6.1 mg/dl. In addition, the patient developed proteinuria (urine protein to creatinine ratio [UPC] 1.28) and microscopic hematuria. The patient underwent renal biopsy and skin punch biopsy. Histopathologic findings revealed pauci-immune crescentic glomerulonephritis with renal granuloma (Fig. 3) and leukocytoclastic vasculitis of the skin. The test result for antineutrophil cytoplasmic antibody (ANCA) against proteinase 3 was positive (3.8 IU/ml; reference range <2.0 IU/ml), whereas test results for myeloperoxidase-ANCA and antinuclear antibodies were negative. We started methylprednisolone pulse therapy (1 g daily for 3 days) after making a diagnosis of GPA with non-infectious endocarditis, DAH, and RPGN (Fig. 4). However, no clinical improvement was observed. After initiating PE every other day, creatinine levels began to slowly decline, but DAH continued to worsen (Fig. 2B). Based on continued DAH progression, combined rituximab with PE therapy was considered.

We performed PE every 2 to 3 days for 5 consecutive sessions in total. Rituximab (375 mg per square meter of body-surface area) was administered once weekly for 4 weeks. The first dose of rituximab was administered the day after the first PE treatment. Two consecutive sessions of PE were performed after the first and second dose of rituximab, respectively. After the second dose of rituximab was administered, there was a significant decrease in alveolar hemorrhage and blood transfusion was no longer required. Chest X-ray and CT findings improved (Fig. 2C and E). Laboratory data, including serum creatinine and hemoglobin, were also notably improved. Mitral valve vegetation disappeared in follow-up TEE. The dosing schedules of rituximab and PE and the patient's clinical course according to therapy are summarized in Fig. 4A. The patient's general condition continued to improve dramatically over the following days.

The patient was discharged with a prescription of prednisolone 50 mg/day, azathioprine 50 mg/day, and trimethoprim/sulfamethoxazole (for pneumocystis pneumonia prophylaxis). Creatinine was 0.7 mg/dl and Hb was 9.2 g/dl on discharge. Prednisolone was tapered to 10 mg/day in the outpatient clinic. Azathioprine was changed to tacrolimus with or without mycophenolate mofetil (MMF) because of persistent mild proteinuria. However, GPA remained stable with low-dose prednisolone and immunosuppressants over a 5-year follow-up period. However, after the 5-year follow-up, rituximab was re-administered (375 mg/m^2^ once per week, for 4 weeks) as creatinine increased to 1.2 mg/dl and UPC rose to 1.21. MMF was discontinued to prepare for pregnancy and renal function remained stable at the 6-year follow-up (Fig. 4B).

## 3 Discussion

While there are several reports on the concurrent use of rituximab and PE to treat other autoimmune diseases,^[6,7]^ there is no standard regimen for this combination, including the dosage of the drug, the order of administration, and the interval between doses. The efficacy and safety of rituximab plus concurrent PE after kidney transplantation have previously been shown.^[8]^ However, no previous reports have discussed this combination for treating GPA, except one case of *de novo* ANCA-associated vasculitis which developed after kidney transplant. In the *de novo* ANCA-associated vasculitis case, contrary to our case, the additional PE had a therapeutic effect when the serum creatinine continued to rise despite rituximab therapy.^[9]^

Our patient presented life-threatening complications of GPA, including hemorrhagic colitis, non-infectious endocarditis, DAH, and RPGN, in a short period of time. In particular, acute renal failure and DAH have been associated with an increased risk of early mortality.^[10]^ Due to the rapid progression of the disease, we considered more aggressive treatments to achieve clinically significant improvement. Another consideration was that the patient, a young woman, wanted to preserve her fertility and refused intravenous cyclophosphamide pulse therapy. As expected, renal function started to improve after initiating PE. However, DAH continued to deteriorate despite treatment with PE, and for this reason combination therapy with rituximab was considered. We cannot exclude the possibility that the patient's recovery could have been obtained by rituximab monotherapy. However, we also cannot exclude the possibility that the cessation of PE may have allowed the re-accumulation of the pathologic antibody, resulting in a rebound phenomenon. As a result, we decided to use concurrent treatment with rituximab and PE and achieved rapid clinical improvement.

PE can remove all solutes in the plasma, including drugs. Therefore, a major concern of this combination therapy is that rituximab may be removed during PE. It is possible to eliminate rituximab and reduce its clinical efficacy if PE is performed shortly after rituximab administration. It has been reported that 50% of rituximab was removed when it was administered <3 days prior to PE.^[11]^ As such, some authors recommended infusing rituximab 48 to 72 hours before the first PE treatment.^[12]^ However, in our case, 2 sessions of PE were done within 24 to 96 hours after the first and second dose of rituximab, but clinical improvement became far more pronounced after the second dose of rituximab. This may be because rituximab had a faster therapeutic effect before it was removed by PE. Rituximab binds to its target as soon as it is administered and immediately initiates cytolysis to induce effective B-cell depletion within 4 days.^[13]^ A study in macaques showed that rituximab administration depleted peripheral blood circulating CD19+/CD20+ cells within 24 hours.^[14]^ Due to the rapid effect of rituximab we therefore assumed that PE did not interfere with rituximab's immunosuppressive effects and could also provide an additional therapeutic effect by removing residual harmful antibodies.

Concurrent therapy with rituximab and PE can be considered in severe GPA refractory to standard therapy, which rapidly progresses to a life-threatening condition, or in young patients who wish to preserve fertility. However, no substantial evidence or treatment guidelines exist regarding the optimal dosing schedule of rituximab plus concurrent PE treatment. This case study suggests that the use of rituximab and concurrent PE may represent a promising combination for severe and refractory GPA. However, further studies are needed to confirm the efficacy and optimal dosing schedule for this combination therapy.

## Author contributions

**Conceptualization:** Yeon-Ah Lee.

**Data curation:** Sang Wan Chung.

**Supervision:** Yeon-Ah Lee.

**Writing – original draft:** Ran Song.

**Writing – review & editing:** Yeon-Ah Lee.

Yeon-Ah Lee orcid: 0000-0001-8007-9131.
